# Inferring Human Colonization History Using a Copying Model

**DOI:** 10.1371/journal.pgen.1000078

**Published:** 2008-05-23

**Authors:** Garrett Hellenthal, Adam Auton, Daniel Falush

**Affiliations:** 1 Department of Statistics, University of Oxford, Oxford, United Kingdom; 2 Department of Biological Statistics and Computational Biology, Cornell University, Ithaca, New York, United States of America; 3 Department of Microbiology, Environmental Research Institute, Cork, Ireland; University of Chicago, United States of America

## Abstract

Genome-wide scans of genetic variation can potentially provide detailed information on how modern humans colonized the world but require new methods of analysis. We introduce a statistical approach that uses Single Nucleotide Polymorphism (SNP) data to identify sharing of chromosomal segments between populations and uses the pattern of sharing to reconstruct a detailed colonization scenario. We apply our model to the SNP data for the 53 populations of the Human Genome Diversity Project described in Conrad et al. (Nature Genetics 38,1251-60, 2006). Our results are consistent with the consensus view of a single “Out-of-Africa” bottleneck and serial dilution of diversity during global colonization, including a prominent East Asian bottleneck. They also suggest novel details including: (1) the most northerly East Asian population in the sample (Yakut) has received a significant genetic contribution from the ancestors of the most northerly European one (Orcadian). (2) Native South Americans have received ancestry from a source closely related to modern North-East Asians (Mongolians and Oroquen) that is distinct from the sources for native North Americans, implying multiple waves of migration into the Americas. A detailed depiction of the peopling of the world is available in animated form.

## Author Summary

Humans like to tell stories. Amongst the most captivating is the story of the global spread of modern humans from their original homeland in Africa. Traditionally this has been the preserve of anthropologists, but geneticists are starting to make an important contribution. However, genetic evidence is typically analyzed in the context of anthropological preconceptions. For genetics to provide an accurate and detailed history without reference to anthropology, methods are required that translate DNA sequence data into histories. We introduce a statistical method that has three virtues. First, it is based on a copying model that incorporates the block-by-block inheritance of DNA from one generation to the next. This allows it to capture the rich information provided by patterns of DNA sharing across the whole genome. Second, its parameter space includes an enormous number of possible colonization scenarios, meaning that inferences are correspondingly rich in detail. Third, the inferred colonization scenario is determined algorithmically. We have applied this method to data from 53 human populations and find that while the current consensus is broadly supported, some populations have surprising histories. This scenario can be viewed as a movie, making it transparent where statistical analysis ends and where interpretation begins.

## Introduction

According to current models, modern humans arose in Africa and spread around the world, with little or no genetic contribution from the hominid populations that they displaced [1],[2],[3]. Genetic diversity decreases progressively with land distance from East Africa [4] providing support for a “serial dilution” model in which diversity was lost progressively in sequential bottlenecks associated with small founder population sizes as new territories were colonized [5],[6]. However, the good fit of serial dilution models might principally reflect recent admixture, which will tend to smooth diversity clines. Numerous questions remain about how many independent bottlenecks occurred as new continents were colonized, the exact land routes involved, and whether there have been genetically important migrations that do not conform to a model of progressive outward expansion [7],[8],[3].

Statistical inference of colonization history represents a considerable challenge. A reasonably detailed description would include (1) the times of major population splits, (2) the effective sizes of each distinct population and/or a list of major bottlenecks and (3) times of major admixture events, when previously distinct populations met and the contributions of the distinct populations to the new hybrid population. Even a complex population based history does not fully describe migration patterns, since isolation by distance can also be important. DNA is passed down through generations in linear segments whose boundaries are determined by meiotic crossovers. Modeling the segment-by-segment inheritance of genetic material is technically challenging even assuming simple demographic scenarios [9]. Adding modern and ancient population subdivision makes computations more complex and introduces the problem of choosing amongst a very large number of possible split and merger scenarios.

We take an approach that models the segmental pattern of human inheritance and also allows comparison between numerous distinct historical scenarios. The approach is predicated on populations arising in an order that can be inferred from the data. For any given ordering of the populations in the sample, we use the copying-with recombination model of Li and Stephens [10] to reconstruct all of the chromosomes. Different orderings of the populations can be compared based on the overall likelihood of generating the entire set of chromosomes in the sample.

Since all the data we analyze is from contemporaneous samples, the assumption of an ordering is incorrect if interpreted literally. However, under a serial dilution model, for example, it is natural to think of populations arising sequentially during radiation from Africa. Subseqent migrations and admixture have complicated this picture but a sufficient signal of these early events remains that the ordering our approach generates can for the most part be interpreted reasonably easily. For example, the “Out of Africa” bottleneck has left a signal of greater genetic diversity in Africans, both at the nucleotide [11] and haplotype levels [12] in the great majority of African and non-African populations, whatever their subsequent demographic history. One of the properties of the Li and Stephens model is that the likelihood of an ordering will generally be higher if the most diverse haplotypes are created first. Our analysis finds the same strong signal that is evident in the summary statistics of diversity; for the dataset of Conrad et al [12] the likelihood of generating two populations, one of which is African, is always higher if the African population is first.

**Figure 1:**
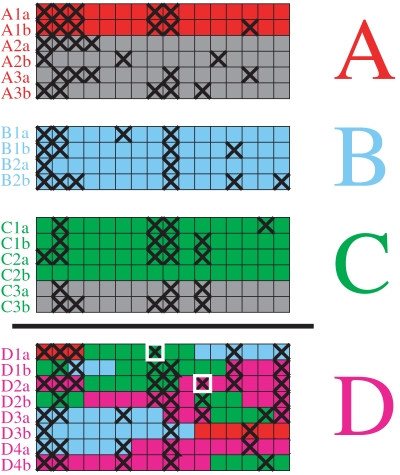
Formation of a new population using its donor pool. The donor pool for population D consists of the red, blue and green haploids from populations A, B and C. Gray haploids are not used as donors. Haploids within population D are created in order, and previously created haploids from population D can be used in the formation of each new one (magenta). For example, haploid D1a is copied from A1a, C2b and B2b, while haploid D1b is copied from C1b, B2b, C1a and D1a. One of the two alleles at each locus is indicated by a black cross, with differences from the copied haploid, i.e. mutations, indicated by a white box around the mutated nucleotide.

In addition to the order in which populations were founded, we would also like to learn about patterns of ancestry. For each new population, a subset of individuals from the previously formed populations is designated as a “donor pool.” In the model, each new haploid genome or “haploid” is formed by copying chromosomal segments from the donor pool or from previously created haploids in the same population (for notational simplicity we assume that every individual consists of two haploids that each contain one of the two copies of the 22 autosomes). The model allows different donor pool combinations to be compared according to the likelihood of generating all the chromosomes in the new population. The number of individuals from each population in the donor pool with the highest likelihood provides an indication of the relative importance of different ancestral sources. For convenience, we refer to the donors using the labels of the modern populations they come from, but they in fact represent surrogates for the shared common ancestors of the donor and recipient populations. The generation of individuals from a single population is illustrated for a hypothetical example in Figure 1.

## Results

### Simulated Data

**Figure 2:**
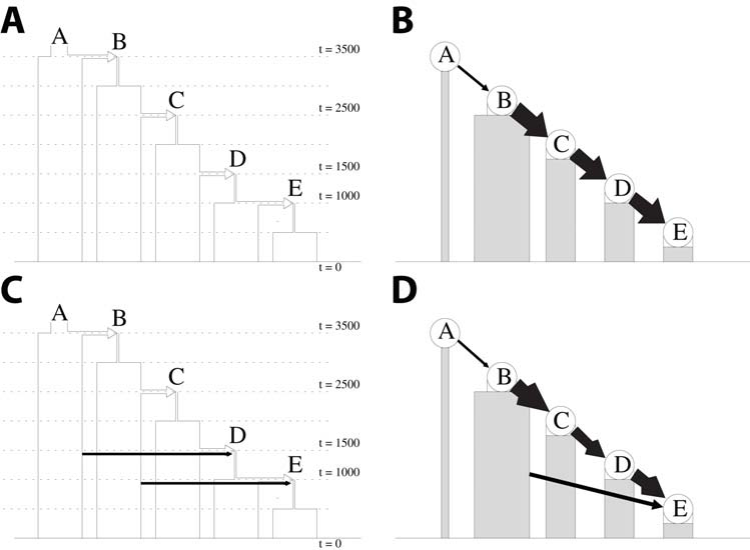
Simulations description and results. (a) and (c) A graphical representation of the simulation parameters. The initial colonization times for each of populations *B*-*E* are denoted with dashed lines, with the times *t* provided on the right in units of generations. Each rectangle represents the demography of one of populations *A*-*E*, as labeled, with the rectangle width scaled by the population size at time *t*. Each arrow represents the sources of colonization for populations *B*-*E*, pointing from source population to sink population, with arrow widths pointing into *D* and *E* roughly proportional to the proportion of genetic material coming from that source. (b) and (d) A graphical representation of typical examples of the results of our model applied to the simulated data, showing inferred ordering and sources for each population (black arrows). The widths of the rectangles are proportional to the number of sampled individuals for each population, and the thickness of the arrow shafts indicate how many of those chromosomes act as donors for subsequent populations.

We tested our inference method using data simulated under a coalescent model [13],[14], with individuals sampled from five populations, labelled A-E, that were generated by sequential bottlenecks (Figure 2-(a)). Parameters were guided by previous demographic estimates [15], with the first bottleneck approximately corresponding to the “Out of Africa” event. In 10 independent realisations of the same scenario (5 with simulated recombinational hotspots, 5 without), the model correctly inferred both the order in which the populations were founded and which populations gave rise to each new one (Figure 2-(b)) and did not infer any additional, spurious sources of ancestry. We then complicated the model by giving populations D and E ancestry from two sources (Figure 2-(c)). The model continued to infer the correct ordering for the formation of the populations and correctly identified the single sources for populations B and C and the two sources for population E in every case. However, in 7 of the 10 simulations, the ancestry of population D was inferred incorrectly, with the model either failing to include population A as an ancestor (as shown in Figure 2-(d)), mistakenly including population B, or both (Table S1). We conclude that, at least for relatively simple scenarios, the model provides an accurate indication of historical relationships between populations but does not always correctly identify minority sources of ancestry, in particular when admixture is ancient.

One potential confounding factor in SNP data is ascertainment bias. The SNPs that are chosen for genotyping are often ascertained based on a limited sample of individual who come from one or a small number of ethnic groups (typically Europeans). For example, in the data of Conrad et al., heterozygosity of the SNPs was actually highest in the Middle East, Central and South Asia and Europe, although these populations are known to be less diverse than Africans. Our method reconstructs haplotypes and therefore we expect it to depend principally on patterns of haplotype sharing and diversity, which a priori should be less sensitive to the ascertainment protocols of individual SNPs. Indeed, in the data of Conrad et al., the haplotype diversity is highest in Africans [12].

In order to test for an effect of ascertainment bias, we performed inference in two extreme ascertainment schemes: one in which we selected SNPs for all populations based only on those that were heterozygous in population *C*, and one in which we selected SNPs for all populations based only on those that were heterozygous in population *E*. The former might represent ascertainment based only on European or Middle Eastern populations. The latter would represent an even more extreme and biased ascertainment, such as ascertaining SNPs using only native Americans. We used 10 of the simulations described above (the ones without recombination hotspots). In 9/10 cases, results were not discernably different from those based on using all SNPs. In the remaining simulation, population B and C were swapped in the inferred ordering under both ascertainment schemes. We conclude that even extremely biased ascertainment has a modest effect on inference.

Our results might also be confounded by the incomplete nature of the sample and by the many complexities of human population history. We have performed additional simulations in order to assess how complications to the scenarios shown in Figure 2 would affect inference. We first evaluated the effect of leaving a population out of the simulated datasets (population D). For all four simulations (two as illustrated in Figure 2-a, one with and one without recombination “hotspots,” and two as illustrated in Figure 2-c, one with and one without recombination “hotspots”), population *C* was chosen as a significant donor population for *E*. Remaining inference was correct (i.e. no other spurious donors were detected, and for the simulations illustrated in Figure 2-c, the model picked up the additional contribution from population *B*.) This is what is expected: with the appropriate donor population missing, our model chooses as its replacement the population that contributed the majority of genetic material to the missing donor population.

**Figure 3:**
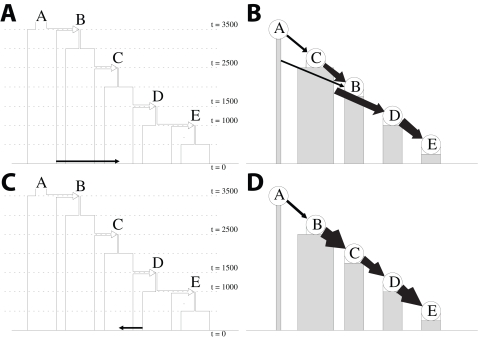
Description and results for simulations with recent admixture. (a) and (c) A graphical representation of the simulation parameters, comparable to Figure 2-a, with the addition of recent migration from population *A* into *C* and recent back migration from population *D* into *C*, respectively. (b) and (d) A graphical representation, as described in Figure 2, of typical examples of the results of our model applied to the simulated data. The recent back migration from population *D* into *C* does not significantly alter inference, while recent migration from population *A* into *C* results in mistakingly inferring that population *C* is a source for *B* in this example.

Complex patterns of admixture might considerably complicate inference. We modified the scenarios shown in Figure 2-a and Figure 2-c by adding recent admixture, either from D to C or from A to C. Examples are shown in Figures 3-a and 3-c. A genetic contribution from population D to C had little effect on inference in 10 different simulations (Figure 3-d). These results show that “back admixture”, for example migrations into Africa, will generally not be detectable by our method. In this simulated example at least, the back admixture did not affect the rest of the inference. The effect of a recent contribution from population A to population C was more substantial. In 5/10 cases (four for the scenario shown in Figure 3-a) the inferred order of populations B and C were swapped (Figure 3-b). The swapping of the populations leaves the genetic connections between the populations correct but inferences on which are sources and which are sinks are confused by the multi-layered migrational history.

### Data of Conrad et al

**Figure 4:**
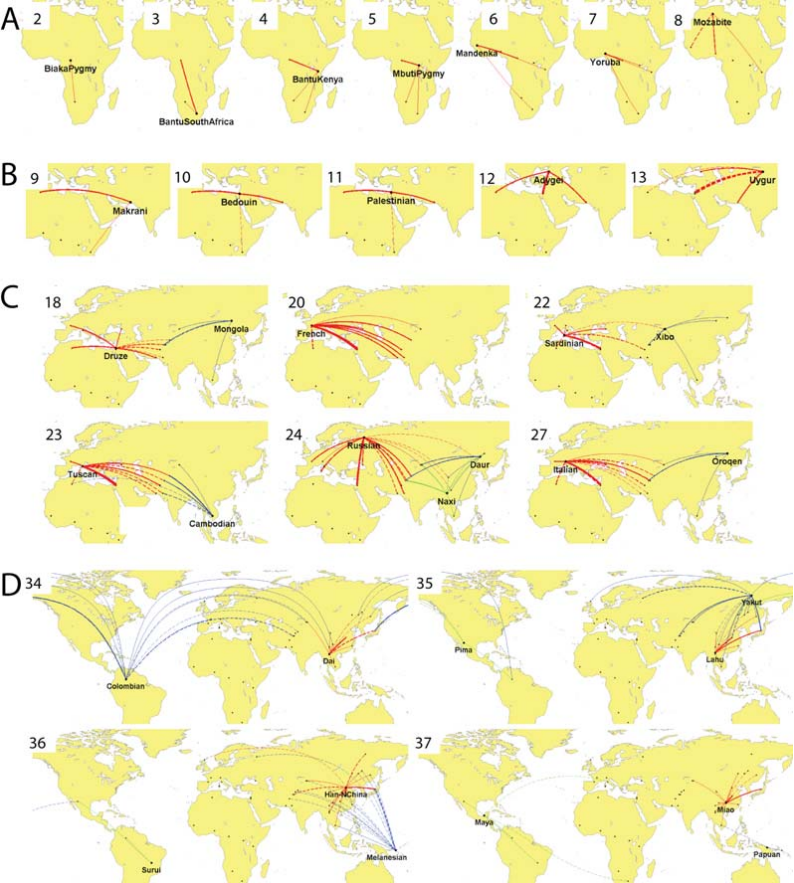
Summary of inferred history of the peopling of the world. The formation of 53 populations has been condensed into 38 frames, shown in full in Movie S1, by displaying the formation of each population as soon as all of its donors are present. When 2 or more populations are formed in the same frame, the connections from their donors are shown in different colors. (A) Africa subsequent to San, (B) initial colonization of central Eurasia, (C) initial colonization of Far East and Europe, (D) Americas and Pacific Islands. The thickness of each line is proportional to the mean estimated number of donor individuals from each source (numerical values provided in Table S2). Solid lines indicate that all or nearly all of the individuals in the population were used as donors. Dashed lines indicate that on average between four individuals and the number of available individuals minus two were used.

We used the same approach to infer the order of birth and ancestral sources of the 53 populations in the Human Genome Diversity Panel using the data from 2,540 linked SNPs across 32 autosomal regions genotyped by Conrad et al [12]. The highest likelihood scenario is shown in Figure 4 and Movie S1. By visually inspecting these results, we have identified nine phases in the colonization of the world. This subdivision is subjective and the phases should not be thought of as occurring strictly in chronological order. For example, East Asia and Europe are peopled almost independently, making their relative position in the ordering nearly arbitrary. Furthermore, Melanesia has multiple sources that reflect ancient and recent migrations that introduced very distinct genetic material (see [16] for a review). Its inferred place in the ordering reflects the most recent of these migrations. Nevertheless, the phases do reflect progressive outward expansion, analogous to that implied by serial dilution models.

1. Sub-Saharan Africa. The first population in the ordering are the San, who are hunter gatherers that live in Southern Africa. Before the Bantu expansion over the last 3,000 years, the ancestors of the San occupied most of Southern Africa, but they have been progressively displaced and currently are restricted to a few pockets [17]. The San contributed ancestry to the next four populations (the Biaka Pygmies, Bantu from South Africa and Kenya, and Mbuti Pygmies) but none subsequent to that. The Bantu are inferred to have contributed to each subsequent African population.

2. North Africa. The Mozabites are the only African population in the sample from above the Sahara. In our analysis, they are the 8^th^ and final African population to arise and are also distinctive because they represent the first population that uses less donor individuals (46 from the Mandenka, Yoruba, and Kenyan Bantu) than their predecessor the Mandeka, who used 64 donors from four populations. We interpret the smaller number of donors as evidence for a bottleneck in the history of the Mozabites, that is not shared by the other African populations in the sample. The small number of donor populations implies that only a subset of the human populations present at the time of the bottleneck contributed to the Mozabite lineage.

3. Central Eurasia. There is no clear pattern to the order of colonization of central Eurasia, with the initial Central Asian populations (Makrani, Uygur) interspersed with those from the Near East (Bedouin, Palestinians) and the eastern edge of Europe (the Adygei). All of these populations have Mozabites as donors, with the first three populations also using Kenyan Bantu. For these three, all 28 Mozabite individuals were used in generating each of the three populations, making it possible that some of the Bantu chromosomes would have been replaced by additional Mozabites or other North or East Africans if they were present in the sample. Overall, non-African populations can each trace approximately 3/4 of their ancestry via the Mozabites (Movie S2, Table S4). The total number of donors increases progressively from 39 for the Makrani to 141 for the Adygei. The high interconnectedness of these populations presumably reflects the absence of region-specific bottlenecks and/or multiple episodes of gene flow between Eurasian populations subsequent to the initial colonization event(s).

4. Central Europe. Aside from the Adygei, the first European populations to arise are the French, Tuscans, and Italians. These three populations have an average of 260 donors, including those from the Mozabites and several Near Eastern and Central Asian populations. This is a larger number than for any non-European population in the sample and highlights the diverse sources of European ancestry.

5. Pre-Han East Asia. The first 8 East Asian populations (Cambodia, Mongolia, Oroqen, Xibo, Yi, Tu, Daur, Naxi) have 50-84 donors, including all 32 individuals from two central Asian populations, the Uygur and the Hazara (except the Tu who use 24/32). This represents an entirely distinct source of ancestry from European populations, who each receive less than 10% of their ancestry via the Uygur and almost none via the Hazara (Movie S2, Table S3). The only other external donors are the Pathan (contributing 12 chromosomes to Mongolians) and the Burusho, Sindhi and Mozabites, who contribute 23, 15, and 4 donors to the Cambodians respectively. We interpret the paucity of donors and the consistence of ancestry patterns as evidence for a single East Asian bottleneck.

6. The extremities of Europe. The final four European populations (the Sardinians, Russians, Orcadians and Basque) all lie on the extremities of the continent. As well as having many European donors, these populations also have a large number from the Near East and Central Asia, consistent with Europe absorbing multiple waves of migrants. The Russians have 375 donors, more than for any other population, including from the Yi, Tu, and Mongolians, indicative of admixture with Far-Eastern populations. The Basque have 4 Hezhen donors but are otherwise similar to other Europeans.

7. The Han expansion. The Han receive their ancestry exclusively from other East Asian populations (including the more westerly Xibo) and represent a principal source of ancestry for several subsequent populations that also have principally East Asian ancestry (She, Japanese, Dai, Lahu, Han from Northern China, and Miao).

8. The Americas. The Colombians are the first Amerind population. 47% of their ancestry can be traced via the Hazara, which is marginally less than typical East Asian populations such as the Han (54%) or Xibo (59%) (Movie S2, Table S3). However, within the descendents of the putative EastAsia bottleneck, their donor pool is diverse, implying that none of the populations in the sample provides a good proxy for the original group or groups that crossed the Bering straight. The Colombians also have French donors, which may reflect post-Colombian admixture. The second American population, the Pima, represents the first North American population. As well as using all 7 Colombians as donors, it uses 8 Mongolians and 4 Oroquen. Neither of these populations acted as donors to the Colombians, suggesting distinct colonization events from different sources. Subsequent American populations did not have any non-Amerind donors, except for the Mayans who have Bantu and Tuscan donors, presumably due to post-Columbian admixture [18].

9. Pacific Islands. All but two of the East Asian populations that donate to the Colombians also donate to the Melanesians, and the Japanese are again the most numerically important with 20 donors. However, the Melanesians have several additional sources of ancestry. These include three populations which are products of the East Asian bottleneck (Oroquen, Han, and Pima), in addition to Central Asian populations (Burusho and Brahui) and Russians. Three Mozabite donors are also estimated, which falls slightly below our conservative threshold for significance (Methods). In total, the Melanesians trace 38% of their ancestry via the Hazara, which is less than East Asian or Amerind populations and implies independent sources of ancestry. The Papuans receive ancestry only from Melanesians and Cambodians, suggesting a shared common bottleneck.

One concern for this dataset is that the number of individuals varies widely among populations (from 6 to 45). We investigated whether this might have a substantial effect on our results by correlating the number of individuals in each population with both its position in the inferred ordering (Figure S1) and the total number of donors it received (Figure S2). Using simple linear regression, no strongly significant correlation was found in either case (p-value > 0.05).

## Discussion

We have inferred a scenario for the peopling of the world using SNP data from 53 populations (Movie S1) by maximising a single likelihood function (Equation 4, see “Ancestry model” section) that uses the extensive information on ancestry provided by linkage between markers in the same chromosomal region. Heuristic algorithms were needed in order to search the very large space of possible scenarios for a high likelihood solution (Methods) but the the scenario was generated automatically and without the use of geographical information apart from population labels. Because our model is simplified, this scenario should not be interpreted as a full chronological colonization history; automatic inference of such a history will require further methodological advances. Nevertheless, because the scenarios our model generates can be related to histories in a reasonably straightforward and transparent fashion, our method is of immediate use in independent hypothesis generation. We describe two such hypotheses below.

These hypotheses gain plausibility because our model also regenerates hypotheses that are already well established in the anthropological genetics literature. First, our results suggest a single major “out-of-Africa” bottleneck. The African populations are all generated prior to all of the non-African ones. Further the great majority of the ancestry of non-Africans goes via a single African population, the Mozabites. The only exceptions are Kenyan Bantu contributions to the first three non-African populations, and South African Bantu contributions to the Sindhi and the Maya. Admixture with descendents of the slave trade can explain the Bantu contribution to the Maya and possibly also to the Sindhi, who have coexisted with a small ethnically African minority, the Sidi, for several hundred years [19]. There is no evidence of any ancient contribution to non-African humans that are independent of the main source populations.

Second, our results are broadly consistent with serial dilution and the peopling of the Americas via the Bering Strait. East Asians arise from central Asians, as do Native Americans. Melanesians have broader ancestry pool than East Asians, suggestive of multiple independent waves of colonization [16]. Their late position in the ordering reflects the ancestry they have derived from East Asians, while the Cambodians precede all other East Asian populations consistent with earlier migrations towards the South [8]. European populations all have a strikingly diverse set of donors, consistent with admixture during “demic diffusion” of near-Eastern DNA into Europe during the spread of agriculture [20] and [21], and the many other documented migrations into Europe, such as from North Africa [22]. Russians have the most diverse sources of ancestry, including from East Asians, consistent with admixture in the sprawling Russian empire.

### Independent Sources of Ancestry for Northern and Southern Amerinds

In our inferred scenario, Pima are the first North American population in the ordering and receive ancestry from the first South American population, the Colombians. The Pima have two additional donor populations, the Oroquen and Mongolians, both of whom reside in Mongolia and neither of which are donors to Colombians. This result is intruiging because it suggests independent sources for North and South Americans and hence multiple waves of migration into the continent, contradicting the current consensus based on available data [23].

We tested the robustness of this inference by swapping the two populations in the ordering and re-inferring donors using the same protocol. The Pima replaced their Colombian donors with a small number of East Asians who were donors to the Colombians (4 donors each from Naxi and She), but the Mongolians and Oroquen remained majority donors. This result mirrors what is found in our simulations; if a donor population is missing (or also present in insufficient numbers in the sample) then it will typically be replaced by one or more of its own donors. The Colombians gained the Pima and lost a substantial number of other donor populations, but kept several from populations that did not contribute to the Pima in either ordering (Daur, Hezhen, Xibo and Burusho).

These results are consistent with substantial gene flow between North and South America but also imply that these have not been strong enough to overwhelm a clear signal of independent colonization. These results also suggest a geographically and historically very plausible scenario: The populations colonizing North East Asia whose members crossed the Bering Strait and whose descendents eventually reached South America were replaced by a population more closely related to modern East Asians (and specifically modern Mongolians). This population subsequently also crossed the Bering Strait and contributed substantially to the ancestry of North American Amerinds. This second wave of migration provides an explanation for the relationship between distance from Siberia and genetic similarity to Siberians [23], which was previously attributed to serial dilution [23]. It also explains why an analysis of the population structure of the Pima and two South American populations based on genome-wide SNP data, using the admixture model of STRUCTURE [24], inferred that the South American populations had a single source of ancestry but the Pima had received approximately half of their ancestry from a second, additional source [25]. Simulation results have shown that the admixture model of STRUCTURE can be surprisingly successful in detecting ancient admixture, even in the absence of source populations, if the number of markers used is sufficiently large [26].

### Gene Flow from Europe to East Asia around the Arctic Circle

In our inferred scenario there is little gene flow between East Asian and Europeans and the Yakut is the only East Asian population to have two European donors; the Russians and the Orcadians. The Russian contribution is not surprising because the Yakut live in North East Russia. The Orcadian contribution is particularly noteworthy because removing these donors reduces the log-likelihood of generating the Yakut chromosomes by 2.5 times more than removing donors from any other population (Table S2). The Orcadians are also the only other European population to donate to other East Asians, namely the Han from Northern China and the Hezhen, who are also amongst the most Northerly East Asian populations in the sample. On this basis we hypothesize that there has been an episode of gene flow from Europe to East Asia. We tested the robustness of this inference by putting Orcadians last in the ordering. The Yakut replaced the Orcadians with Sardinians, who are a major donor to the Orcadians. The Hezhen and the Han from Northern China did not acquire new European donors, consistent with the gene flow from Europe being less quantitatively important to these two populations than to the more Northerly Yakut. Orcadians did not gain any East Asian donors by being placed last in the ordering, strengthening the inference that the direction of the gene flow was from Europe to East Asia.

Our results provide evidence for two continent-scale bottlenecks, the first affecting non-Africans and the second affecting East Asians, with both groups having a small number of donors from outside the region. Unfortunately, the limitation of both our method and the sampled populations make it difficult for us to make detailed inferences about the nature of these bottlenecks. Most of the ancestry of non-Africans comes via the only only North African population in the sample, the Mozabites, who are also the last African population to be formed. However, their intermediate position might reflect back migration from the Middle East and/or Europe[27],[28],[29]. Simulation results suggest that our method is likely to miss this type of back admixture. Indeed, if Mozabites are allowed to receive ancestry from any populations and not only those that precede them in the ordering, they get approximately 70% from these two regions, consistent with the results of STRUCTURE for the same populations [30]. In any case, a much better sample of East and North African populations would be required to elucidate the nature of the bottleneck.

A similar problem of interpretation occurs for the East Asian bottleneck. A majority of the ancestry of East Asians comes via two central East Asian populations, the Uygur and the Hazara. However these populations could have come to resemble East Asians through back migration. Indeed, if these populations are placed last in the ordering, then more than 40% of their donors are East Asian. If donors for the East Asian populations are inferred while excluding the Uygur and the Hazara from the dataset, the first populations have a somewhat larger number of donors from a wider range of Central or West Asian populations (Brahui, Makrani, Balochi, Sindhi and Adygei) than shown in Movie S1, but populations later in the ordering revert to having predominantly East Asian donors, supporting a strong East Asian bottleneck that contrasts with the wide sources of ancestry of Europeans.

The major simplification of our model is to assume that the populations were founded in an order. Since the DNA samples came from living humans, the ordering does not reflect age, but instead bottlenecks and admixture events that distinguish more recently formed populations from older ones. Complexities in human history make this ordering somewhat arbitrary. For example, the Melanesians have been founded by multiple waves of migrations. Their position late in our ordering reflects the substantial proportion of their ancestry that comes from East Asians. However they also have other, independent sources of ancestry that reflect migrations that are likely to predate those that gave rise to the modern East Asian populations. Information on the timing of different waves of migration could potentially be obtained from more extensive DNA sequence datasets by examining the sizes of the blocks of DNA that are inherited from different donor populations. Recent admixture would result in individuals sharing large contiguous segments from particular donor populations [26],[31]. Recent shared ancestry would result in individuals receiving large contiguous segments from particular donor haploids.

A fully realistic history would avoid any ordering of the modern populations. One potential avenue for extending the current approach to achieve this goal would be to impute chromosomes from “ancestral populations,” which would both represent populations that existed in the past and also act as efficient donors for the modern haplotypes. Generation of such populations poses a number of statistical and computational challenges but could potentially allow a chronological, multi-layered history to be inferred. Accurate reconstruction of historical migrations depends crucially on the use of appropriate samples and any geographical interpretation can be confounded by major population movements. Further, it should ideally be demonstrated that the results are robust to which parts of the genome are used in analysis. Further methodological innovation and genome-wide SNP datasets from diverse human populations [25],[32] should allow unprecedented detail in the reconstruction of the ancestry of extant humans.

## Materials and Methods

### Genotype Data

We used the 32 autosomal regions in Conrad et al [12], each of which consisted of approximately 80 biallelic SNPs across 330 kilobases of the genome. SNP data were collected for a total of 927 individuals sampled from 53 different populations, with sample sizes ranging from 6 to 45 individuals per population. Data were kindly provided to us as haplotypes, which were phased using fastPHASE [33] on each region as previously described [12].

### Ancestry Model

Li and Stephens [10] described a likelihood based model that captures the principal features of the genealogical process with recombination while remaining computationally tractable for large datasets. Under the model, the chromosomes are generated in order, with chromosomes being copied segment-by-segment from those earlier in the ordering. In our notation, every individual consists of two haploids, each consisting of a single phased haplotype per genotyped region. The *L* total SNPs in each haploid are listed one region at a time, in order within each region.

Suppose that we wish to generate a particular haploid h_*_, using *j* pre-existing donor haploids *h*_1_,…,*h_j_*. Let *ρ* represent the crossover recombination rate per unit physical distance across the genome, assumed fixed. The conditional probability Pr(h_*_ | *h*_1_,…,*h_j_*; *ρ*) is structured as a Hidden Markov model, where the hidden state *X_l_* represents the existing haploid from the set *h*_1_,…,*h_j_* that haploid h_*_ copies from at each site *l*  =  1,…,*L*. The switches in copied-from haplotype are modelled as a Poisson process with rate *ρ/j*. The transition probabilities for *X* between sites *l* and *l*+1 are as follows:
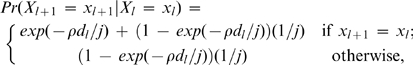
(1)where d*_l_* is the physical distance between SNPs *l* and *l*+1. If *l* and *l*+1 are on separate genetic regions, we set *d_l_  = * ∞. The observed state sequence component of the Hidden Markov Chain, the probability of observing a particular allele given the haploid that h_*_ is copying from at a given SNP, allows for “imperfect” copying that depends on a per site mutation parameter 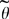:
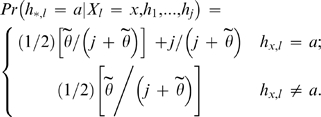
(2)


Here *h_j,l_* refers to the allelic type of haploid *j* at SNP *l*. The mutation parameter 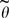 is fixed, as in [10], as Watterson's estimate with one expected mutation event per site, i.e. 
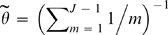
 [34] for *J* total haploids. To calculate Pr(h_*_ | *h*_1_,…,*h_j_*; *ρ*), a summation is performed over all permuations of the copying process, i.e. a summation over all possible *x*, which can be accomplished efficiently using the forward algorithm (e.g. [35]). In the analyses presented here, we used an alteration of (1) above, using the “PAC-B” version described in [10].

Note that the probability of recombination events (i.e. switches) and mutations goes down as the number of haploids *j* increases. This mirrors a key property of data generated under the coalescent, that the probability that a segment from an additional chromosome will be identical by descent with a segment from chromosomes 1…*j* increases with *j*. This property also means that different orderings will have different likelihoods that at least in part reflect the demographic history of the individuals in the sample. For example, if a subset of individuals in the sample have a particularly high level of diversity, then the overall likelihood will generally be higher if these individuals are generated early rather than late in the ordering.

In previous implementations of the Li and Stephens algorithm, it has been assumed that each new haplotype is made using all previous haplotypes. This leads to the formula for the probability of observing *J* haploids, conditional on *ρ*:
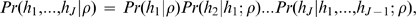
(3)where 
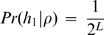
 as in [10].

However, in the context where individuals come from differentiated populations, a higher likelihood may be obtained by using only a subset of the pre-existing individuals as donors. In order for a donor individual to increase the likelihood of generating h_*_, there needs to be chromosomal segments, whether large or small, that are more similar to h_*_ than any of the others in the donor pool. Individuals from populations that are more differentiated from h_*_ than others in the donor pool are likely to contain few such segments. Further, every individual increases the value of *j* by 2, and for each segment that is copied a 1/*j* term appears in the likelihood, corresponding to choosing amongst the *j* donor haploids. Thus the presence of differentiated individuals in the donor pool can decrease the overall likelihood.

Here we are interested in investigating ancestry at the population level. We therefore make some assumptions about orderings and donors that are justifiable if the individuals within each population share the same demographic history. In practice, population labels are initially defined based on geographic and ethnic criteria, and the degree of homogeneity within the labelled populations can be assessed on multilocus genetic data [18]. These assumptions considerably reduce the computational complexity of the problem. Within each population, haploids are assumed to be generated – and donors are used in generating them – in the order they appear in the input file. In generating a set of haploids *H* across *K* populations, we further assume that:

1. The *K* populations are generated in sequence according to an order of colonization *U*  =  (*u*_1_,…,*u_K_*), where *u_k_* denotes the *k* ^th^ population in the order. To simpify notation, we subscript each population by its position in the ordering, with 1 representing the initial population and *K* the final population to be colonized.

2. Each population *k* has a fixed set of donor individuals from previous populations in the order, *D^k^*. The membership of *D^k^* is determined by *k*−1 integers, 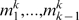, reflecting the number of individuals from previous populations 1,…, *k*−1 that donate genetic material to population *k*.

3. Within a population *k*, haploids are made in order using the previous haploids as donors, i.e. for 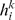, the *i* ^th^ haploid genome of population *k*, the total donor pool 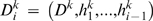.

4. The formation of each population *k* involves a single genome-wide recombination rate, *ρ_k_*.

Let 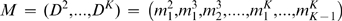 represent the number of donor individuals from populations 1,…,*K*−1 for each of populations 2 to *K*, and let Φ  =  (*ρ*_1_,…,*ρ_K_*) denote the set of recombination rates involved in forming all populations *K*. Then the probability of the haploid data of all populations, *H*, conditional on *U*, *M*, and Φ, is:
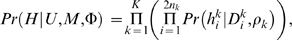
(4)where *n_k_* denotes the number of individuals in population *k*.

We want to maximise (4) across all possible orderings of populations, donor sets and recombination rates. This represents a very large search space. We used a hill climbing approach and some MCMC updates to find a good solution.

We first set out to generate an inital order of colonization, *U* ^(0)^, using a pairwise analysis. For each of the *K*(*K*−1) permutations of pairs of populations, we calculated the probability of forming all haploids in both populations using (3). Specifically, for each pairwise combination, we calculated (3) twice, once using a haploid ordering where all of one population's haploids are formed first and the other where they are formed last. For each calculation, we maximized over *ρ* using 200 iterations of Markov Chain Monte Carlo (MCMC). In particular, for each MCMC iteration *r*, a new proposal of log_10_ *ρ*, log_10_ *ρ* ^(*r*)^, was selected from a uniform(−1,1) distribution shifted to be centered on the previous value of log_10_ *ρ*. This new value of *ρ* was then accepted or rejected via a Metropolis-Hastings step, i.e. *ρ* ^(*r*)^ was accepted with probability min(*a*1), where 
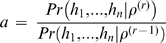
, or otherwise rejected. (Here we take a uniform prior on log_10_ *ρ* between −7 and 3.) We compared the final probability values at *r*  =  200 for each of the two orderings, awarding 1 point to the population that was first in the highest likelihood ordering. Our initial ordering *U* ^(0)^ was based on the number of points received by each population, with the highest scoring population considered the first population formed. Ties were broken randomly; in the data of Conrad et al [12], there were nine instances where two populations had the same number of points and two instances where three populations had the same number of points (Table S5).

We calculated the likelihood for *U* ^(0)^ and for subsequent orderings using a greedy algorithm for each *k* to obtain values of *M* and Φ. For each *k*, first (4) was evaluated using all possible individuals as donors, i.e. 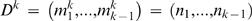, maximizing over *ρ_k_* using 200 iterations of MCMC as described above, giving 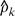. Next, the change in the likelihood obtained by setting 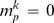, fixing 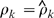, was evaluated for each 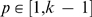 for which 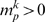. If each of these changes decreased the likelihood, the algorithm stopped. Otherwise, for the *p* which resulted in the highest increase in likelihood, 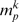 was set to 0 and *ρ_k_* was re-maximised conditional on this new value of *D^k^* using a further 200 MCMC iterations, and the algorithm continued.

We used an iterative procedure to obtain orderings with progressively higher overall likelihood. Specifically, for each 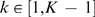, we calculated the likelihood of the ordering *U* ^*^  =  (*u*_1_…,*u_k_*_+1_,*u*_k_,…,*u_K_*). In each iteration, we accepted all changes in ordering that increased the likelihood or left it the same, the only exeptions being where two or more such changes were incompatable with each other. In these cases, we accepted those changes that improved the likelihood the most. This procedure was repeated until the changes either decreased the likelihood or reversed a change that had previously been made. For the data of Conrad et al [12], 13 such iterations were performed, providing the ordering *U* ^(13)^ (Table S5). The overall log-likelihood improved by 344 in these 13 iterations. For the simulated data, no changes in ordering were accepted. For the data of Conrad et al [12] but not the simulated data, we performed an analogous procedure to generate *U* ^(14)^ but comparing all possible conFigure urations of triplets of orderings, i.e. *U* ^*^  =  (*u*_1,_…,*u_k_*,*u_k_*_+1_,*u_k_*_+2_,…,*u_K_*), *U* ^*^  =  (*u*_1,_…,*u_k_*,*u_k_*_+2_,*u_k_*_+1_,…,*u_K_*), *U* ^*^  =  (*u*_1,_…,*u_k_*_+1_,*u_k_*_+2_,*u_k_*,…,*u_K_*), *U* ^*^  =  (*u*_1,_…,*u_k_*_+1_,*u_k_*,*u_k_*_+2_,…,*u_K_*), *U* ^*^  =  (*u*_1,_…,*u_k_*_+2_,*u_k_*,*u_k_*_+1_,…,*u_K_*), and *U* ^*^  =  (*u*_1,_…,*u_k_*_+2_,*u_k_*_+1_,*u_k_*,…,*u_K_*). We accepted 4 such changes, improving the log-likelihood by a further 67. We then recalculated new optimal values of *ρ_k_* for this ordering, which improved the log-likelihood by a further 60, and checked pairwise population swaps based on these new values. None of the proposed swaps increased the likelihood further, so this gave us our final ordering 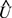 (Table S5).

The greedy algorithm assumes that for each population *k*, the preceeding populations contribute either all or none of their chromosomes to the donor pool *D^k^*. In order to find a solution which allowed fractional contributions from donor populations, we used an MCMC approach, conditional on this final ordering 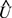 and final values of *ρ_k_*, 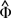. Let 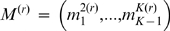 be the donor pool at iteration *r*, with 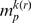 the number of donor haploids from population *p* to population *k* at iteration *r*. Initially, we set 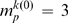 for all 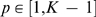 and 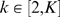. We then performed the following steps at each iteration *r*  =  1,…,*R*:

1. randomly choose one of *k*'s donor populations 1,…,(*k*−1) with uniform probability; call this population *p*

2. randomly choose 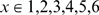 with uniform probability

3. if *r* is an even number, set 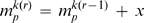


4. if *r* is an odd number, set 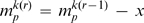


5. if 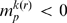 or 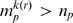 then reject the change, i.e. 
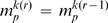
.

6. Otherwise, accept the change with probability min(*a*,1), where 
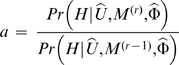
.

For both the simulated data and our application to the data of Conrad et al [12], contributions were deemed significant if the average number of donors exceeded 2. For our simulated data, we used the results of a single MCMC run with 5,000 iterations, including 1,000 Burn-in iterations. For the data of Conrad et al [12], different MCMC runs converged on slightly different local optima, as a result of the complexity of the search space. We therefore used a consensus of the results of the greedy solution and two independent MCMC solutions. For each MCMC run, we initially ran the algorithm for 10,000 iterations, including 2,000 iterations of burn-in. In 17 cases (both runs of Japanese, Lahu, Maya, Pima and Papuan and one run of Dai, Italian, Sardinian, Surui, Tujia, Karitiana and She) the algorithm initially got stuck in a local optimum but then jumped to a significantly better solution (>30 improvement in log-likelihood) after the burn-in was complete. We therefore continued the run for a further 10,000 iterations, using the last 8,000 to estimate the posterior. In each of these 17 instances no further large improvement in likelihood occurred during these 10,000 iterations, indicating convergence on a local optimum. The consensus (Table S2) included donor populations that were significant in at least two of the three solutions and for which the number of donor individuals, averaged across the three solutions, was also greater than 4.

## Simulations

To test our method's performance under simple demographic scenarios, we performed several sets of simulations using the coalescent-based simulation software msHOT [14],[13]. In particular, we performed simulations under a sequential bottleneck model, using five populations and four bottleneck events (Figure 2). We performed simulations for two different scenarios, as shown in Figure 2-a and Figure 2-c. For both scenarios, the population size of *A* beyond *t*  =  3500 generations is 10,000 chromosomes, bottleneck size is 2,000 for the initial colonizations of each of *B*-*E*, and present-day population size is 25,000 for *A*-*E*. In the simulatons with admixture, *D* has 75% contribution from *C* and 25% from *A*; *E* has 75% contribution from *D* and 25% from *B*. The number of sampled individuals ranged between 6 (for population A) and 45 (for population B). This range was chosen to match that found in the data of Conrad et al [12].

For each scenario, we performed ten independent simulations. In each we simulated 32 genetic regions of size ≈330kb and 80 SNPs for each population. We considered two different models of recombination (five simulations under each model). The first model consisted of a constant recombination rate *ρ_sim_* across all 32 regions, with *ρ_sim_*  =  1.0/kb. Here *ρ_sim_*  =  4*N*_0_ *c*, where *c* is the rate of crossover recombination as before and *N*_0_ is the present-day population size of each population, i.e. *N*_0_  =  25000. This rate closely matches the observed average rate of recombination in humans, assuming a present-day population size of 25,000. The second model included recombination *hotspots*, or narrow areas of the genome with intense recombination activity relative to the surrounding region. For the latter recombination model, hotspot parameters were chosen to mimic current observations on typical hotspot characteristics [36],[37],[38],[39]. The number of hotspots was selected from a Poisson distribution such that they occured genomewide every 40kb on average. Each hotspot's width in kilobases was sampled from a uniform(1,2). Its intensity *λ*, or relative rate of recombination compared to regions outside of hotspots, was sampled such that log_10_^λ^∼Uniform(1.0,2.5). This intensity distribution restricts hotspots to have recombination rates between 10-316 times that of background rates, with 50% of hotspots expected to have intensities between 10 and 32. Outside of hotspots, the rate of recombination in all regions was fixed at *ρ_sim_*  =  0.2325/kb. Finally, we imposed an additional restriction that hotspots had to be at least 5kb apart in a region. These parameters resulted in a genomewide average recombination rate of ≈1.0/kb, with 15 maximum hotspots per region and roughly 78% of the total recombination occuring in the 3.7% of the sequence genomewide designated as hotspots. These numbers match – or are slightly more extreme than – current observations [38].

After simulating the haplotypes for each region based on the above parameters using msHOT, SNPs were randomly chosen to mimic allele frequencies present in the data of Conrad et al [12] in the following manner. The 0^th^, 10^th^, … , 90^th^, and 100^th^ quantile values of SNP allele frequencies for all populations combined were found for the Conrad et al [12] data across all regions. SNPs were then selected in the simulated data such that, for 80 total SNPs per region, ≈10% were between the 0 and 10^th^ quantile values of the real data, ≈10% were between the 10^th^ and 20^th^ quantile values of the real data, etc. Histograms of the allele frequencies of our simulated data after ascertaining in this manner were roughly comparable to that of the data of Conrad et al [12] (data not shown).

The data we analyzed consisted of haplotypes estimated by the authors of Conrad et al [12] using the program fastPHASE [33]. Therefore we used fastPHASE to estimate the haplotypes of our simulated data after selecting SNPs based on the ascertainment strategy described above. That is, we pretended the haplotype information from the msHOT simulations was unknown and phased the genotype data using fastPHASE v.1.2.0 on each region, for each of the five simulated populations separately. We used roughly the same fastPHASE parameters as [12], using *H*  =  500, *T*  =  20, and *C*  =  25, with *K*  =  20 clusters for populations with more than 40 haplotypes and *K*  =  10 clusters otherwise (see the fastPHASE documentation for a full description of these parameters and [12] for a full description of their phasing strategy).

For the scenarios with recent forwards or backwards admixture, recent admixture was added such that 0.25% of the “sink” population was comprised of new migrants from the donor population each generation, starting 20 generations ago and continuing until present-day. Otherwise the simulations were the same as those based on Figure 2-a and Figure 2-c described above (five for each under each recent admixture scenario), without recombination hotspots.

## Supporting Information

Figure S1 Number of individuals per population versus our model's inferred ordering. Note that there is no clear correlation between the two.
(0.003 MB PDF)

Figure S2 Number of individuals per population versus our model's inferred total number of donors. Note that there is no clear correlation between the two.
(0.003 MB PDF)

Table S1 Results of simulations shown. Shows inferred order and mean number of donor individuals contributed from donor to recipient. Contributions are treated as significant if more than two individuals on average are inferred as donors. Red indicates inference of a genuine source, orange indicates inference of a genuine source that highlights the recent admixture, blue indicates a genuine source that is not inferred, green indicates an incorrect source that is inferred, and purple indicates an incorrect swap in the ordering.
(0.06 MB XLS)

Table S2 Summary of results for Conrad et al [12] dataset. Shows the mean number of donors for each of the sources shown in Movie S1 and also totals. The last column shows the reduction in log-likelihood by excluding the population in the greedy solution.
(0.06 MB XLS)

Table S3 Ancestry of particular populations. For each recipient population, gives proportion of donor chromosomes that went via each existing population. Values were estimated recursively, working backwards from the labelled population to the first (San) by assuming that the amount of genetic material passed on by each population was proportional to the number of donor individuals it contributed.
(0.09 MB XLS)

Table S4 Ancestral routes for particular populations. Shows values for particular lines as shown in Movie S2.
(0.68 MB XLS)

Table S5 Inference of ordering. First column gives score for each population based on pairwise comparisons. The second to fifth columns give details of the initial ordering chosen based on those scores (U(0)), including the inferred value of the recombination rate *ρ* and the likelihood of generating the haploids for each population, both of which are calculated based on the greedy approach. For subsequent iterations, *ρ* is kept fixed, but the likelihoods for particular populations change. After the final iteration, new *ρ* values are calculated for each population, shown in the final column of the table.
(0.04 MB XLS)

Movie S1 Inferred history of the peopling of the world. Donors are listed at the bottom in order according to the mean number of individuals that are used. See Figure 4 for further details. Numerical values are given in Table S2.
(0.94 MB CDR)

Movie S2 Inferred history of chromosomes for individual populations. Each frame shows the path that chromosomes took from their origin in Southern Africa in reaching the population labelled in each frame. The width of each line indicates the proportion of the chromosomes that travelled by that route, with the diameter of the circle indicating the total proportion of chromosomes that went via that location (diameter of San  =  1.0). Values were estimated recursively, working backwards from the labelled population to the first by assuming that the amount of genetic material passed on by each population was proportional to the number of donor individuals it contributed. Numerical values are given in Table S3 and Table S4.
(1.23 MB CDR)
