# Correction: Inferring Human Colonization History Using a Copying Model

**DOI:** 10.1371/annotation/da6e20fe-f8eb-4e44-8669-6c20c7102b3d

**Published:** 2008-06-04

**Authors:** Garrett Hellenthal, Adam Auton, Daniel Falush

There was an error in the penultimate sentence of the Abstract. The correct sentence is: Native North Americans have received ancestry from a source closely related to modern North-East Asians (Mongolians and Oroquen) that is distinct from the sources for native South Americans, implying multiple waves of migration into the Americas.

