# Supplementary figures and images for: Inferring Human Colonization History Using a Copying Model
Source: PLoS Genet. 2008 May 23;4(5):e1000078. doi: 10.1371/journal.pgen.1000078 (PMC2367454; doi:10.1371/journal.pgen.1000078)

## Number of Individuals vs Total Number of Donors

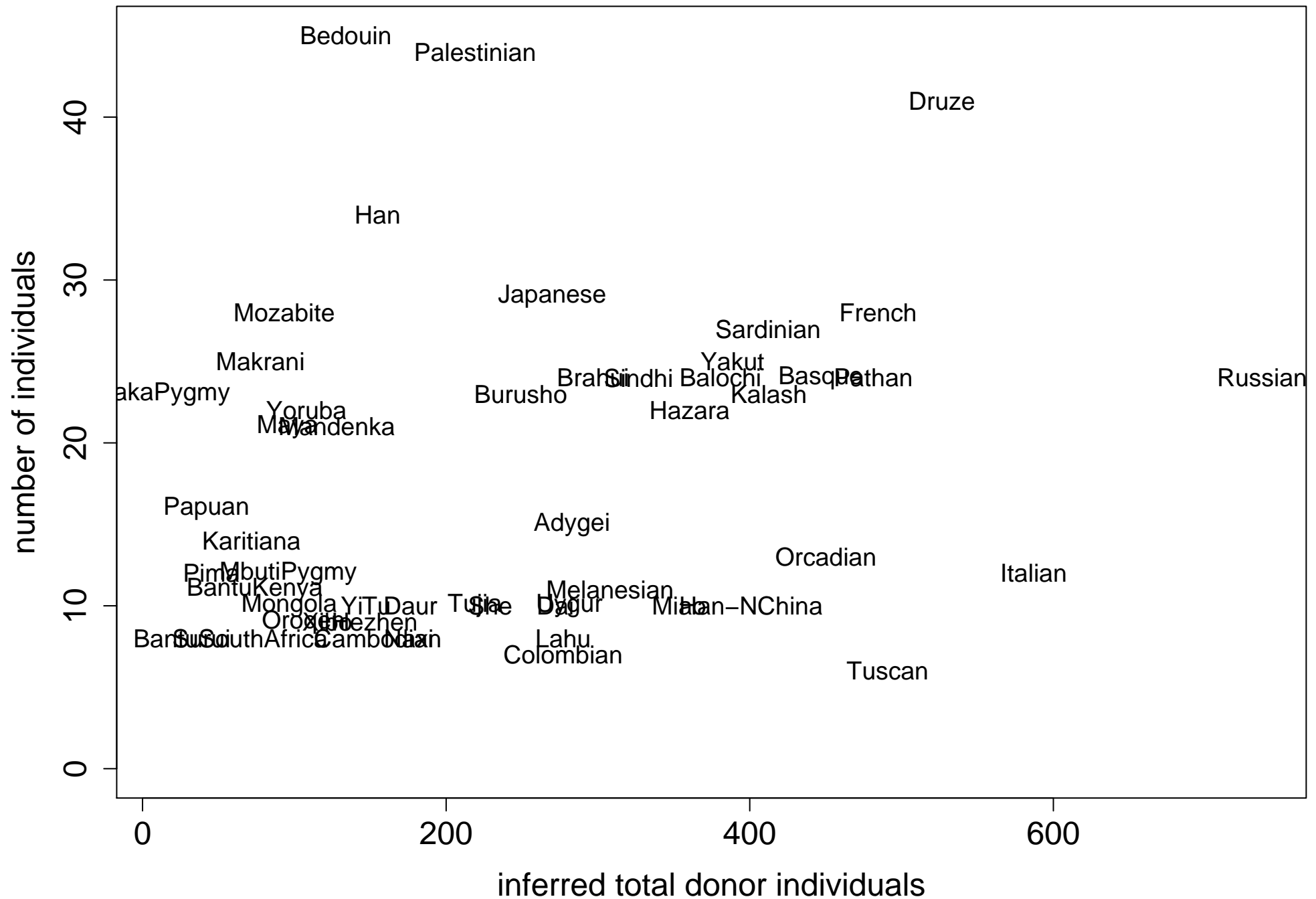

Supplement: Figure S1 — Number of individuals per population versus our model's inferred ordering. Note that there is no clear correlation between the two. (0.003 MB PDF) [file pgen.1000078.s001.pdf]

# Number of Individuals vs Inferred Order

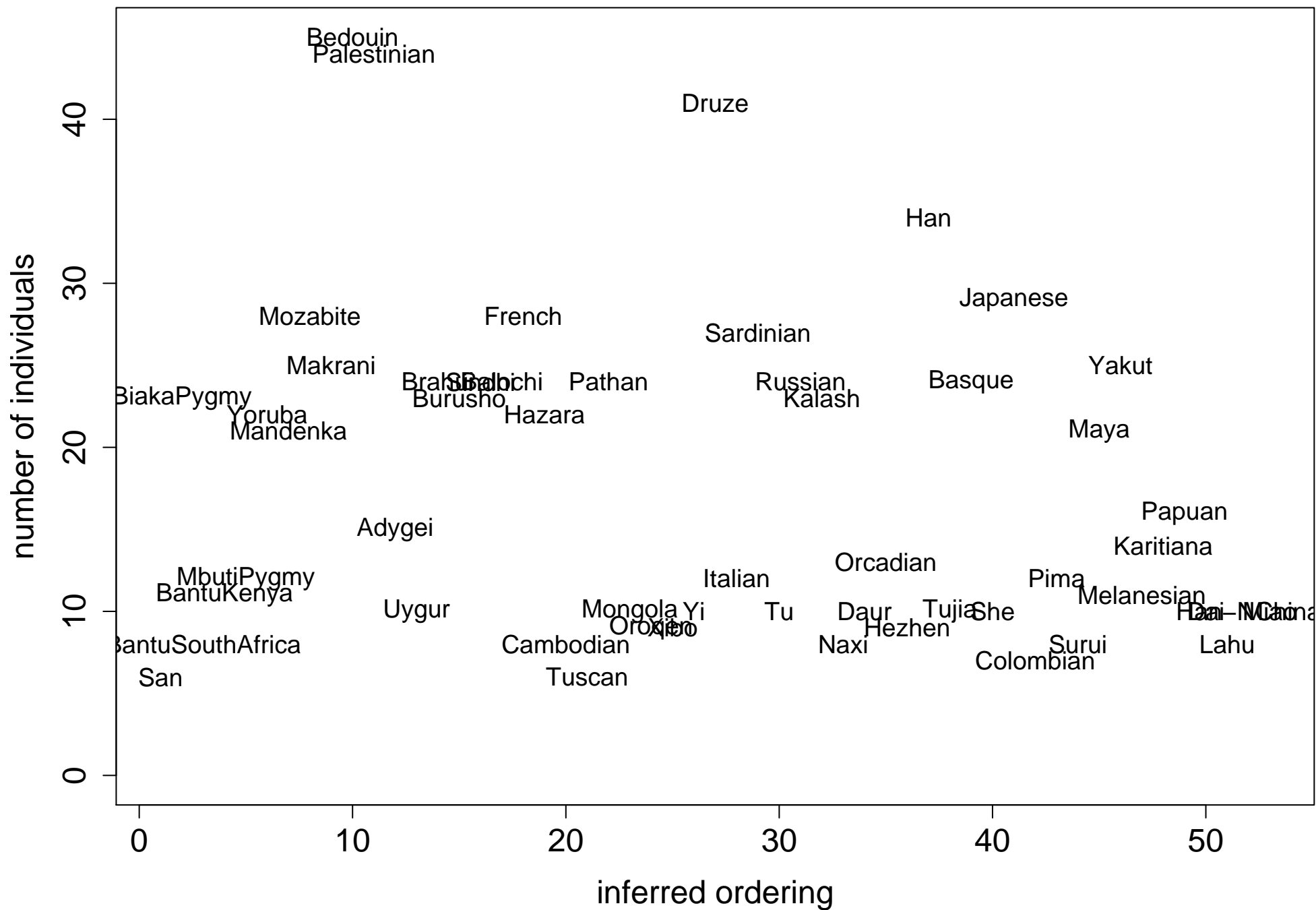

Supplement: Figure S2 — Number of individuals per population versus our model's inferred total number of donors. Note that there is no clear correlation between the two. (0.003 MB PDF) [file pgen.1000078.s002.pdf]
